# Hidden secrets of the cancer genome: unlocking the impact of non-coding mutations in gene regulatory elements

**DOI:** 10.1007/s00018-024-05314-z

**Published:** 2024-06-20

**Authors:** Sandra Iñiguez-Muñoz, Pere Llinàs-Arias, Miquel Ensenyat-Mendez, Andrés F. Bedoya-López, Javier I. J. Orozco, Javier Cortés, Ananya Roy, Karin Forsberg-Nilsson, Maggie L. DiNome, Diego M. Marzese

**Affiliations:** 1 Cancer Epigenetics Laboratory at the Cancer Cell Biology Group, Institut d’Investigació Sanitària Illes Balears (IdISBa), Palma, Spain; 2 Saint John’s Cancer Institute, Providence Saint John’s Health Center, Santa Monica, CA USA; 3 International Breast Cancer Center (IBCC), Pangaea Oncology, Quiron Group, 08017 Barcelona, Spain; 4 Medica Scientia Innovation Research SL (MEDSIR), 08018 Barcelona, Spain; 5 Faculty of Biomedical and Health Sciences, Department of Medicine, Universidad Europea de Madrid, 28670 Madrid, Spain; 6 Department of Immunology, Genetics and Pathology and Science for Life Laboratory, Uppsala University, Uppsala, Sweden; 7 University of Nottingham Biodiscovery Institute, Nottingham, UK; 8 Department of Surgery, Duke University School of Medicine, Durham, NC USA

**Keywords:** Enhancers, Promoters, Insulators, Silencers, Single nucleotide polymorphisms, Single nucleotide variants

## Abstract

Discoveries in the field of genomics have revealed that non-coding genomic regions are not merely "junk DNA", but rather comprise critical elements involved in gene expression. These gene regulatory elements (GREs) include enhancers, insulators, silencers, and gene promoters. Notably, new evidence shows how mutations within these regions substantially influence gene expression programs, especially in the context of cancer. Advances in high-throughput sequencing technologies have accelerated the identification of somatic and germline single nucleotide mutations in non-coding genomic regions. This review provides an overview of somatic and germline non-coding single nucleotide alterations affecting transcription factor binding sites in GREs, specifically involved in cancer biology. It also summarizes the technologies available for exploring GREs and the challenges associated with studying and characterizing non-coding single nucleotide mutations. Understanding the role of GRE alterations in cancer is essential for improving diagnostic and prognostic capabilities in the precision medicine era, leading to enhanced patient-centered clinical outcomes.

## Introduction

The Human Genome Project, which generated the first map of the human reference genome, marked a pivotal milestone in genetics research. However, the significance of non-coding genomic regions, formerly considered “junk DNA,” remained largely unexplored. The convergence of large-scale sequencing technologies and computational biology pipelines in the field of functional genomics has revealed the importance of non-coding regions in orchestrating gene expression programs [1]. These regions, collectively known as gene regulatory elements (GREs) [2], have been classified based on their impact on gene expression into gene promoters, enhancer elements (EEs), insulator elements (IEs), and silencers. Additionally, genomic alterations on GREs, ranging from single nucleotide variants (SNVs) to larger structural variants (SV), can disrupt the expression of regional as well as distant genes in disease states, specifically in cancer [3]. Consequently, these previously overlooked genetic modifications can dramatically impact normal gene expression programs [4, 5] by affecting the binding of transcription factors (TFs) [6], altering genome organization [7], modulating chromatin accessibility [8], or changing regional DNA methylation levels [9] at GREs.

Two main types of mutations that play a pivotal role in various diseases are involved in GRE dysregulation: germline single nucleotide polymorphisms (SNPs) and somatic SNVs [10]. Notably, genome-wide association studies (GWAS) have linked different SNPs located within non-coding regions to various types of cancer [11]. In contrast, projects such as the Pan-Cancer Analysis Whole Genomes (PCAWG) have identified thousands of non-coding somatic SNVs in numerous cancer types [12, 13]. Regardless of the origin, these point mutations are enriched within the transcription factor binding sites (TFBS) of GRE sequences in cancer [14–17]. This area of functional genomics opens an opportunity to leverage the clinical utility of non-coding mutations in different disease states, specifically in the context of cancer, bringing a chance to improve diagnostic, prognostic, and predictive models to improve patient’s clinical outcomes. While the impact of large SV on precision oncology has been discussed elsewhere [5], this review provides an overview of the recent findings on the functional impact of non-coding somatic and germline single nucleotide alterations affecting GREs in cancer. Considering the growing body of evidence highlighting the clinical significance of SNVs within non-coding regions of the genome, there has been a surge of innovation in technologies aimed at their comprehensive characterization and the exploration of their intricate molecular mechanisms [18, 19], which is also discussed.

### Types and definitions of gene regulatory elements

**Fig. 1:**
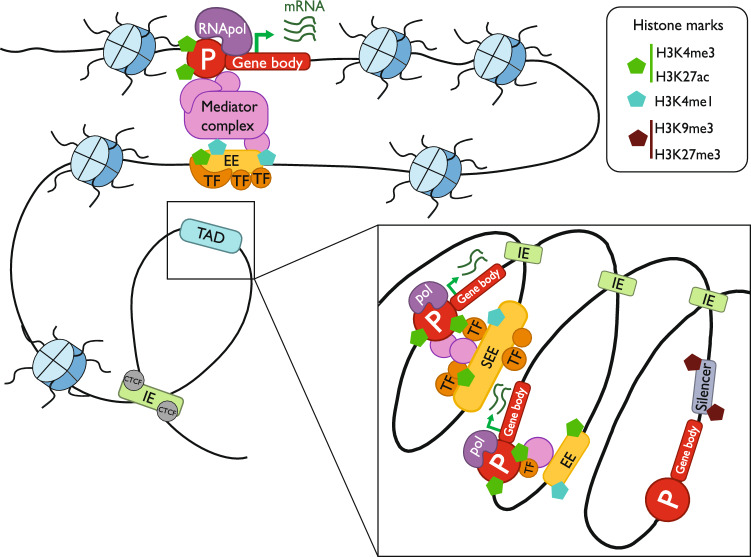
Schematic overview of GREs and their chromatin interactions. The figure shows a DNA strand with promoters (P with red circles), enhancer elements or super-enhancers elements (EEs or SEEs in yellow), insulator elements (IEs in green) with CTCF binding, and silencer element (grey). The representation includes a Topologically Associating Domain (TAD), transcription factors (TFs), mediator protein complex, histones, and their marks in each type of GRE

GREs are defined by a specific combination of histone marks and conglomerates of TFBS [20–23]. Based on the impact on the expression of regional as well as distant genes, GREs are classified into promoters, enhancer elements (EEs), insulator elements (IEs), and silencers (Fig. 1). Regarding the annotation of GRE, it is crucial to acknowledge the work conducted by the ENCODE (Encyclopedia of DNA Elements) consortium, which employed different experimental techniques – including ChIP-seq of TFs and histone marks, RNA-seq, among others – to characterize the regulatory elements in the human genome [24, 25]. This section provides information about each type of GRE to better understand the impact of single nucleotide variations on cancer biology.

**Gene promoters** comprise sequences upstream of the transcription start site (TSS), where the transcription machinery is assembled [26]. Many genes have been described to have alternative TSS [27]; as a result, different promoters can be associated with a single gene. However, the impact of gene promoters is usually associated with a nearby single gene. On the other hand, **enhancer elements** (EE) are defined by clusters of TFBS whose activation may affect the expression of both regional and distant genes by recruiting coactivators in cooperation [28]. Thus, these *cis*-regulatory elements have a highly variable location relative to the target genes [29]. EEs are activated or repressed in a spatial–temporal manner to define cellular fate during development [30]. As a consequence of its activation, the chromatin is looped allowing the proximity between EEs and promoters through the action of mediator proteins called cohesins [31]. Moreover, a single EE can regulate multiple genes, and one gene can be regulated by multiple EEs [32]. In addition, conglomerates of EEs have been defined as **super-enhancer elements** (SEEs). These GREs span large genomic regions and are enriched in binding motifs for master TFs and cofactors [33, 34]. Multiple TFs can occupy SEEs, modulating gene expression through SEE-promoter interactions, and forming core transcriptional regulatory circuits [35]. These elements are capable of driving cell-type-specific genes involved in key hemostatic functions and defining cell fates. Thus, the alteration of SEEs has been demonstrated to be crucial for tumor development and progression, as well as in therapeutic drug resistance or insensitivity [36, 37]. Another type of GREs known as **silencer elements** have the opposite effect compared to EEs. These regulatory elements repress gene expression by blocking the TF aggregation on either the gene promoter or upstream regulatory elements [23, 38]. Moreover, dual-function regulatory elements (REs) have been characterized in *Drosophila* [39], yet their presence in mammals remains unexplored. These genomic regions exhibit the capacity to function as both EEs and silencer elements. Notably, more than 5% of human silencers display regulatory element properties, underscoring the versatility of REs [40]. Finally, interactions between gene promoters and EEs can be influenced by another type of GRE that acts as boundary elements, known as **insulator elements** (IEs) [41]. These types of GREs are responsible for generating and maintaining the chromatin structural units called Topologically Associating Domains (TADs), which divide the genome into different compartments confining the interaction of GREs inside TADs [42]. Thus, alterations affecting IEs disrupt the TAD organization and have also been confirmed to contribute to tumorigenesis [43]. Activation of IEs mainly involves the binding of two critical proteins, CCCTC-binding factor (CTCF) and cohesin (RAD21) [44, 45]. Therefore, dysregulation of IEs alters gene expression programs by reshaping the landscape of promoter-EE interactions. Apart from single nucleotide mutations involving CTCF binding sites, many IEs can be impaired through abnormal DNA methylation [46, 47].

### *Cancer*-associated non-coding single nucleotide mutations in GREs

Numerous SNPs and SNVs have been identified outside of coding genomic regions [48, 49]. Mechanistically, these alterations can influence the stability of GREs, leading to an alteration in the balance between the expression of tumor suppressor genes and oncogenes [50–52]. In this context, genomic alterations that lack measurable biological or phenotypic effects are often referred to as "passenger mutations" [53], whereas mutations conferring advantages to tumors are denoted as "driver mutations". The latter can be further categorized as either "major drivers" or "mini drivers", based on their magnitude of impact [54]. Another important factor in determining the impact of the SNV is the type of GRE affected. Tables 1, 2, 3 highlight the most important SNVs associated with cancer, including both SNPs and somatic mutations that affect promoters, EEs, and IEs, respectively.

**Table 1:** Non-coding mutations in promoter regions with impact on cancer development

Cancer type	Non-coding mutation	Promoter affected	Effect	Ref
Breast cancer	chr14:38064406G > A	FOXA1	Enhances TF binding ability of E2F family, upregulating FOXA1	[79]
Breast/Ovarian cancer	rs117039649	MDM2	Reduces the Sp1 binding affinity Reduces MDM2 expression	[68]
Endometrial cancer	rs2870820	MDM2	This leads to allele-specific expression T allele impairs NF-κB binding ability	[69]
Glioblastoma	chr7:80,552,013; T > C	SEMA3C	Modifies the binding affinity of different TFs increasing SEMA3C expression	[78]
Glioblastoma	chr5:1,295,411; G > A chr5:1,295,433; G > A	TERT	Enhanced GABP recruitment and increased TERT expression	[75]
Liver cancer	chr4:81,187,908; A > T	FGF5	A MYC binding site is generated	[84]
Melanoma	rs2279744	MDM2	Enhances the E2F1 binding affinity Increases MDM2 expression	[66]
	chr5:1,295,228; C > T chr5:1,295,250; C > T	TERT	Facilitates the binding of ETS	[76]
	different C > T transitions	SDHD	Modulates the binding of GABPA, GABPB1, and ETS1	[71, 83]

**Table 2:** Non-coding mutations in EEs and SEEs promoting alterations in TF affinity with impact on cancer development

Cancer type	Non-coding mutation	TFBS affected	Effect	Ref
Lung cancer	rs2853677	Snail1	The SNP breaks the Snail1 binding site and derepresses *TERT* expression promoting lung adenocarcinoma susceptibility	[96]
	rs9390123 rs9399451	POU2F1	Two SNPs in the same EE allow the *PHACTR2-AS1* oncogene transcription to create a new POU2F1 binding site	[89]
Neuroblastoma	rs2168101	GATA	rs2168101 changes *LMO1* expression disturbs GATA binding and promotes neuroblastoma pathogenesis	[103]
Low-grade glioma	rs55705857	OCT2/4	The risk allele breaks OCT2/4 binding, increasing *Myc* expression	[91]
Breast cancer	rs4784227	FOXA1	T allele decreases *TOX3* expression due to an increase in FOXA1 binding affinity. This binding promotes the TLE repressor recruitment, which diminishes the EE strength	[93]
	rs10941679	FOXA1	This SNP interacts with *MRPS30* and *FGF10* promoter regions and changes FOXA1 affinity	[95]
	rs9383590	GATA3	Recurrently mutated EE, which interacts with the *ESR1* promoter, harbors this SNP, promoting breast cancer progression	[94]
Promyelocytic leukemia	chr11:32421395C > A chr11:32421396C > A chr11:32421397G > T chr11:32421397G > A	MYB	Repetitive non-coding somatic and germline at chromosome 11 come together to reduce WT1 expression by interfering with MYB binding and disrupting chromatin looping	[98]
Ovarian cancer	chr6:27,870,735 T > A	TEAD4	Somatic mutations at these EE perturb the PAX8 transcriptional network during the progression of ovarian cancer	[97]
Colorectal cancer	rs6854845	Long-range chromosomal interaction destruction	This SNP perturbs chromosomal interaction between the SE and *EREG*, *EPGN,* and *CXCLs* genes with a role in inflammation response and cell growth	[100]
	rs11064124	VDR	rs11064124 destroys VDR binding and coactivates *CD9* and PLEKHG6 transcription which are tumor suppressor genes	[101]
Melanoma	rs2995264	MEOX2	G-allele diminishes *OBFC1* expression and decreases MEOX2 TF affinity related to cancer initiation	[90]
Diffuse large B-cell lymphoma	rs6773363 rs9831894	CBX5 MYBL2 News long-range chromosomal interactions	With rs6773363, the SE physically interacted with genes related to immune response. But with rs9831894, the SE can establish spatial interaction with oncogenes. The C allele at rs9831894 disrupted the CBX5 and MYBL2 binding motif	[102]
Chronic lymphocytic leukemia	rs539846	RELA	This SNP breaks RELA TFBS and It is associated with *BMF* down-regulation impacting anti-apoptotic BCL2, an oncogenic hallmark	[104]

**Table 3:** Single nucleotide mutations impacting IEs stability on cancer development

Cancer type	Non-coding mutation	TFBS affected	Effect	Ref
Lung cancer	rs60507107	CTCF	rs60507107 is involved in lung tumorigenesis regulating CTCF binding thus repercussing *DAGLA* expression	[107]
	rs58163073	SOX2	T allele increased SOX2 binding affinity suffering a change in chromatin conformation bringing on less distance with the VDAC3 gene, promoting its expression and cancer evolution	[117]
Breast cancer	rs11540855	CTCF	The mutation affects *ANKLE1* expression, associated with breast cancer risk	[108]
	rs7714232	A breast cancer risk EE is attracted	The SNP regulates MAP3K1 expression because it puts in touch with an active enhancer associated with breast cancer risk	[116]
	rs16886272	GATA3	This SNP manages MAP3K1 expression because it is in contact with a DNA fragment that contains a GATA3 binding site	[116]
	rs4752575	FOXA1	rs4752575 alters FGFR2 expression increasing breast cancer susceptibility. This SNP establishes physical interactions with a chromatin fragment that contains multiple TFBSs for FOXA1, highly associated with breast cancer risk	[116]
	rs9940301	3 breast cancer risk EEs are put in touch	This SNP is in contact with a chromatin location that contains 3 putative breast cancer risk EEs. Consequently, these EEs activate CDYL2 gene expression, an oncogene, or a tumor suppressor depending on the tumor type	[116]
Colorectal cancer	rs12263636 rs3740253 rs7071351	New long-distance enhancer-promoter interaction loop	These SNPs may increase *RPS24* expression through enhancer–promoter interaction	[118]
	rs174575	New long-distance enhancer-promoter interaction loop	rs174575 upregulates colorectal oncogene *FADS2* which were mediated by E2F1	[92]
Prostate cancer	rs6152	The SNP forms contact with a prostate cancer risk EE	This EE is associated with prostate cancer because affects AR expression	[116]
	rs6983267	AR	The SNP is in contact with a DNA fragment having an AR binding site that regulates *POU5F1B* gene expression, implicated in tumor cell growth	[116]
	rs721048	An EE with prostate risk is activated	rs721048 forms physical contact with a chromatin fragment in which there is an active EE associated with prostate cancer because it regulates *EHBP1* and *OTX1* expression	[116]
Melanoma	chr5:111,887,319 G > A	CTCF	Mutated CTCF anchor motif promotes disruption of loop formation, promoting APC low expression, a tumor suppressor gene	[109]
Pancreatic cancer	rs2001389	CTCF	The allele G weakened CTCF affinity, and it was related to the lower expression of an antioncogene *MFSD13A* whose knockdown promoted tumor cell proliferation	[119]

### Non-coding single nucleotide mutations within gene promoters

**Fig. 2:**
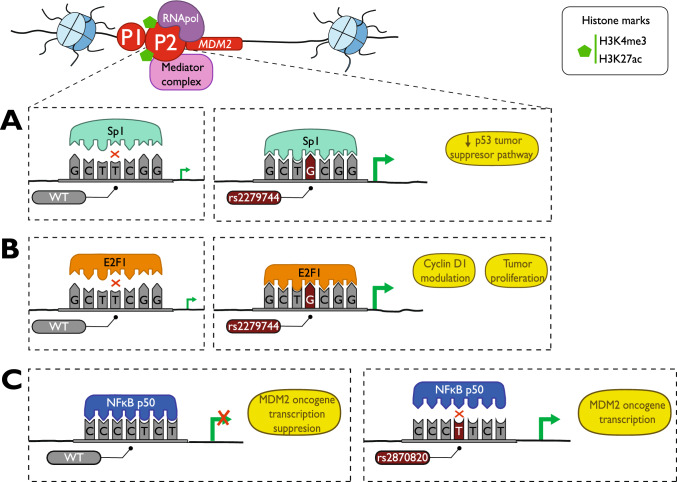
SNPs in *MDM2-P2* promoter and its oncogenic consequences. **A** The presence of the germline alteration rs2279744 promotes Sp1 binding diminishing the p53 tumor suppressor pathway [66]. **B** The same mutation in the MDM2-P2 promoter generates a strong affinity with the E2F1 TF, modulating cyclin D1 and generating tumor proliferation [67]. **C** rs2870820 (SNP55C > T) is related to MDM2-P2 transcriptional activity. SNP55C has an affinity for NFκB p50 homodimers and it suppresses oncogene *MDM2* transcription. However, the alteration does not retain this affinity with NFκB p50 favoring oncogene transcription [69]

SNPs in promoter regions that disrupt the TFBS are studied across various tumor types, including lung cancer [55], hepatocellular carcinoma [56], neuroblastoma [57–59], and breast cancer [60–62]. A well-described example of germline single nucleotide mutations in tumorigenesis are the SNPs located on the promoter region of the oncogene Murine Double Minute 2 homolog (*MDM2*) [63]. MDM2, which is under the control of two distinct promoters, P1 and P2 [64], can negatively modulate the tumor suppressor p53, targeting it for proteasomal degradation [65]. For example, the G-allele of the rs2279744, known as SNP309 at the P2 promoter increases *MDM2* expression by elongating the Sp1 TFBS. This alteration significantly reduces the tumor suppressor p53 levels [66], ultimately enhancing the risk of cancer development in humans, as depicted in Fig. 2A. In the context of melanoma pathogenesis, the SNP309 variation generates a stronger E2F1 binding site (Fig. 2B), which is responsible for cyclin D1 modulation and tumor proliferation [67]. Another germline mutation described within this promoter (rs117039649), located just 24 bp upstream of the SNP309, has the opposite impact, by reducing the Sp1 binding affinity and, therefore, the expression levels of MDM2 in ovarian and breast cancer [68]. Furthermore, a third SNP (rs2870820) found on the MDM2 promoter, known as SNP55, leads to an allele-specific expression by impairing NF-κB binding (Fig. 2C) [69]. Thus, the *MDM2* gene highlights the complex interplay between genetic variations and gene regulation, demonstrating that the same promoter can be affected by different SNPs, causing a substantial differential effect in pathogenesis.

Somatic SNVs have been identified as affecting gene promoters in different cancer types as well [70–72]. One of the most relevant findings was in the human telomerase reverse transcriptase (*TERT*) gene [73, 74]. In glioblastoma, Bell et al. discovered two somatic SNVs (chr5:1,295,411; G > A and chr5:1,295,433; G > A) in the *TERT* core promoter, which led to an enhanced GABP recruitment [75]. In melanoma, the *TERT* promoter contains two highly recurrent somatic SNVs (chr5:1,295,228; C > T, and chr5:1,295,250; C > T) allowing the binding of the ETS TF [76]. The consequence of the increased affinity of these TFs is the reactivation of *TERT*, a common mechanism in multiple cancers that allows bypassing the replicative senescence [76]. Another example is found in the promoter of *SEMA3C*, a gene related to tumor development in glioma stem cells [77]. The presence of a somatic SNV (chr7:80,552,013; T > C) has been found to modify the binding affinity of several TFs, such as RUNX1, ZNF354C, FOXA2, and EN1. Importantly, this mutation alters the binding site for FOXA1 in the *SEMA3C* promoter, leading to a reduced TF binding to the region [78]. Similarly, a somatic SNV in the *FOXA1* promoter region (chr14:38,064,406; G > A) has been detected in primary breast cancers [79]. The mutant motif creates a stronger binding site for TF members of the E2F family, promoting high expression levels of *FOXA1*. This gene works as a transcriptional pioneer factor in breast cancer, enhancing chromatin accessibility for estrogen receptor interaction to its genomic targets [80], and has been linked to decreased response to fulvestrant, an estrogen receptor antagonist [81, 82]. In melanoma, the *SDHD* promoter contains different C > T transitions within the core ETS TF binding motifs, such as C524T and C523T, specifically affecting the binding of GABPA, GABPB1, and ETS1 [71, 83]. These alterations lead to a decreased expression of *SDHD,* which is associated with an unfavorable prognosis [83]. Furthermore, in primary liver cancer, Lowdon RF et al. identified a somatic mutation (chr4:81,187,908; A > T) in the *FGF5* promoter region, which generates a new MYC binding site and enhances FGF5 expression [84]. SNVs at promoter regions affecting gene expression in cancer have been compiled in Table 1.

### Single nucleotide mutations affecting enhancer and super-enhancer elements in *cancer*

Non-coding single nucleotide mutations within EEs and SEEs have been shown to disrupt critical TFBSs and influence transcriptional regulation through intricate interactions between these genetic variations and the epigenomic landscape. GWAS studies have demonstrated this phenomenon across a spectrum of cancer types, including but not limited to ovarian cancer [85, 86], colorectal cancer [87], and chronic lymphocytic leukemia [88], as summarized in Table 2.

**Fig. 3:**
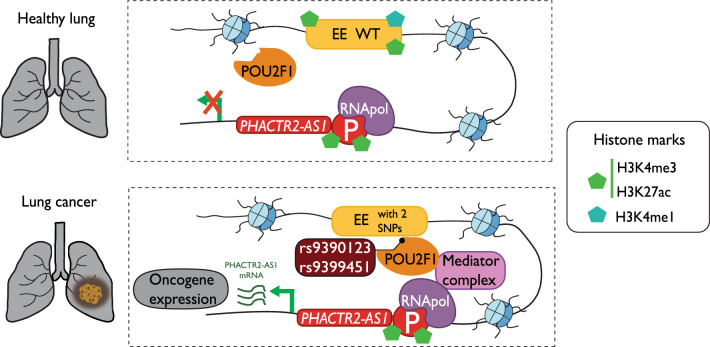
The rs9390123 and rs9399451 are located in the same EE and affect the binding of TF and *PHACTR2-AS1* transcription. In lung cancer, the two germline mutations allow POU2F1 binding in an enhancer element (EE in yellow) and, consequently, the interaction between it and the promoter of the oncogene *PHACTR2-AS1*, favoring its expression [89]

Germline alterations have been shown to have an important role in EE abnormal activity in cancer. For instance, in lung cancer, two SNPs (rs9390123 and rs9399451) were detected within an EE located near the *PHACTR2-AS1* gene, resulting in the creation of a new POU2F1 binding site that potentially modulates the DNA repair capacity of this cancer type (Fig. 3) [89]. Cardinale et al. characterized the role of rs2995264, an SNP located within an EE near the *OBFC1* gene, in melanoma [90]. The presence of the G allele of this SNP reduces the binding affinity of the MEOX2 TF, thereby promoting carcinogenesis. In the context of low-grade glioma, the presence of rs55705857 in a brain-specific EE disrupts the OCT2/4 binding motif. This alteration leads to an abnormally higher expression of MYC by enhancing the interaction between the EE and *MYC* gene promoter [91]. Similarly, the SNP rs174575 exerts its influence on a long-range EE and modulates *FADS2* gene expression through an increased binding affinity for E2F1. The upregulation of *FADS2* leads to an increase in Prostaglandin E2 metabolism, a known oncogenic factor contributing to colorectal cancer development [92].

In breast cancer, SNPs located in EEs have been demonstrated to influence tumorigenic gene expression programs. Notably, multiple breast cancer-associated SNPs exhibit enrichment in FOXA1 binding sites. As previously mentioned, FOXA1 acts as a pioneer factor by binding to highly compacted heterochromatin and exposing genomic areas to other transcription factors, hence influencing cancer-related pathways. In this context, the presence of the [T] rs4784227 allele in an EE leads to an elevated affinity of FOXA1 compared to the [C] reference allele. In vitro experimentation demonstrated that this SNP, which is located 18.4 kb upstream of the *TOX3* gene, interacts with FOXA1/Groucho/TLE proteins, resulting in local chromatin condensation and transcriptional suppression. As a result, the [T] rs4784227 variant allele is found to have a repressive effect on *TOX3* gene expression [93]. Moreover, the rs9383590 SNP impairs the interaction between GATA3 and an EE located upstream of the *ESR1* gene TSS. In this context, GATA3 acts as a repressor and the SNP consequence was an increase in *ESR1* gene expression [94]. Another noteworthy SNP (rs10941679) located within an EE alters the gene expression program of breast cancer cell lines by establishing interactions with the *MRPS30* and *FGF10* promoter regions. This leads to *MRPS30* downregulation, a gene involved in the apoptosis process, and *FGF10* upregulation, a well-known oncogene [95]. In lung adenocarcinoma, Li X et al. characterized another relevant SNP (rs2853677) within an EE near the *TERT* gene, which disrupts the Snail1 TFBS and enhances *TERT* gene expression [96].

Several somatic SNVs have also been identified in EEs. For example, a somatic SNV within an EE converges upon the TEAD4/PAX8-binding sites, leading to the perturbation of the expression levels of PAX8-target genes during the progression of ovarian cancer [97]. Interestingly, somatic and germline mutations can cooperate in favoring TFBS perturbations. For example, in a study on promyelocytic leukemia conducted by Song H et al., recurrent non-coding somatic and germline mutations were detected in an EE located inside the third intron of the *WT1* gene. These mutations were found to reduce the binding of MYB, thereby disrupting the EE-promoter interaction. Consequently, it resulted in a decreased expression of WT1, a critical regulator of hematopoiesis [98].

Interestingly, new data indicates that approximately 64% of disease-associated SNPs are found within genomic regions with SEE activity [99]. One example is the rs6854845, which disrupts long-range chromosomal interaction between SEE and target genes *CXCLs*, *EPGN*, and *EREG*. This has been linked to a transcriptional switch that has a pivotal role in cell proliferation and inflammatory response in colon cancer [100]. Similarly, the rs11064124 G > A influences the binding of the vitamin D receptor (VDR), resulting in reduced expression of the tumor suppressor genes *CD9* and *PLEKHG6*, ultimately promoting the development of colon cancer [101]. In diffuse large B-cell lymphoma, Kleinstern et al. identified two SNPs, rs6773363 and rs9831894, both located in the same SEE. While the presence of rs9831894 leads the SEE to interact with immune response genes, the rs6773363 variant promotes the interaction with oncogenes, consequently fostering tumor growth [102]. In a study of associations between SNPs and neuroblastoma, it has been observed that the rs2168101 G > T disrupts a binding site for the members of the GATA TF family within a SEE involved in *LMO1* gene expression, ultimately contributing to neuroblastoma progression [103]. Finally, in chronic lymphocytic leukemia, the rs539846 variant disrupts a RELA binding site within an SEE. This disruption is associated with decreased expression of *BMF*, thereby enhancing the expression of the anti-apoptotic protein BCL2, a well-known oncogenic hallmark [104].

Alternatively, somatic SNVs can also contribute to generating new SEEs. In a subset of T-cell acute lymphoblastic leukemia cases, a singular somatic alteration has been observed to profoundly affect MYB binding affinity, resulting in the formation of a SEE located upstream of the *TAL1* oncogene [105]. The evidence highlighting the involvement of non-coding mutations in governing SEE is just beginning to emerge. The characterization of non-coding mutations affecting these elements may unveil novel theranostic biomarkers to enhance the management of this disease.

### Single nucleotide mutations on insulator elements in *cancer*

**Fig. 4:**
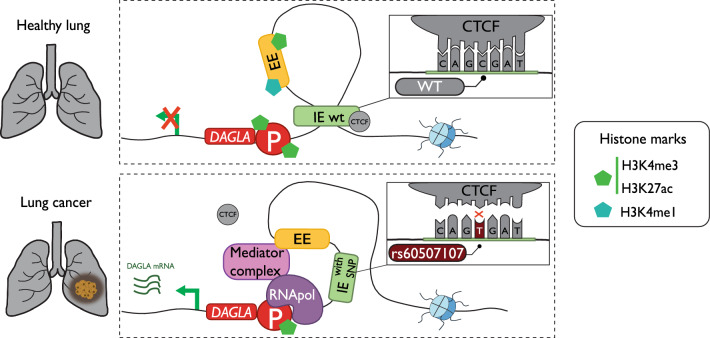
The SNP rs60507107 disturbs CTCF binding affinity in an IE. Lung cancer development is promoted by the presence of this SNP because it breaks an insulator element (IE in light green), increasing *DAGLA* expression, a gene that is related to carcinogenesis in different types of tumors [107]

A comprehensive examination of SNPs and somatic SNVs affecting IEs is summarized in Table 3. As previously discussed, the activation of IEs relies on CTCF binding and the formation of homodimers with other CTCF-IE complexes. Somatic as well as germline single nucleotide mutations that interfere with the consensus CTCF motif can disrupt the binding of the CTCF protein and, therefore impact the activation of IEs [15]. This phenomenon has been observed in various types of cancers [106]. For example, the rs60507107 impacting a CTCF binding site (Fig. 4) has been identified as a susceptible SNP for lung cancer development [107]. An elevated risk of breast cancer development has been associated with the G/G variant of the rs11540855. Functional genomics studies in both tissue and cell lines have revealed that individuals with this variant have higher expression of the *ANKLE1* gene due to the disruption of the CTCF binding to an IE that controls the expression of the *ANKLE1* gene [108].

Similarly, somatic SNVs have been recognized to influence IEs in cancer. In the context of melanoma, a somatic mutation (chr5:111,887,319 G > A) has been identified in a CTCF motif. This non-coding mutation disrupts the loop formation, resulting in the dysregulation of APC expression, a crucial tumor suppressor gene [109]. Another study conducted in melanoma identified an insulator (chr19:41,767,305–41771623) that displayed seven different somatic hotspots. Different somatic mutations on this IE increased the expression of TGFB1, contributing to aggressiveness. A mechanism detailing how UV-induced DNA damage leads to somatic SNVs in CTCF binding sites and, as a consequence, mutagenesis in human skin cells has been promoted [16].

In a separate study in gastrointestinal cancer, Guo YA et al. delved into the prediction and evaluation of three somatic non-coding mutations that have a discernible impact on CTCF binding sites, subsequently causing alterations in TFBSs [110]. Non-coding mutations in CTCF motifs near oncogenes such as *KCNJ5*, *FLI1*, and *MYC* have also been reported in gastrointestinal cancer [111]. Despite their potential implications for cancer development, non-coding single nucleotide mutations affecting IEs are currently relegated to the status of "passenger" mutations [112] and remain overlooked in cancer research.

Furthermore, when CTCF binding is disrupted, it can trigger the upregulation of genes that are typically protected within TADs, isolated from neighboring EEs [113]. Both SNPs and somatic SVs have been observed to interfere with these contact domains. This disruption can result in the activation of oncogenes through the formation of novel promoter-enhancer interactions [114]. In particular, some TADs exhibit SNP-driven alterations in a cancer-specific manner due to the organization of genes known to drive cancer progression [115]. For instance, recent findings by Osman et al. unveiled the presence of risk SNPs at the boundaries of certain TADs in prostate and breast cancer, specifically associated with GREs implicated in these pathologies [116].

In patients with lung squamous cell carcinoma, the presence of the T allele of the rs58163073 variant has been demonstrated to significantly enhance SOX2 binding affinity within the TAD boundary. This alteration in chromatin conformation near the *VDAC3* gene results in an elevated expression that fosters cancer progression [117]. Colorectal cancer exhibits a distinct regulatory scenario, where the upregulation of the *RPS24* gene is driven by the presence of three SNPs within a TAD boundary (rs3740253, rs7071351, and rs12263636). This enables the formation of a pathological promoter-EE interaction [118]. In pancreatic cancer patients, the G allele of the rs2001389 weakens the binding site for CTCF resulting in TAD disruption. This alteration diminishes the expression of the tumor suppressor gene *MFSD13A*, ultimately culminating in increased tumor proliferation [119].

### Silencer elements affected by non-coding mutations

The impact of non-coding single nucleotide mutations on silencer elements remains poorly understood in cancer. While some SNPs have been identified, to date, no somatic SNVs have been reported within silencer elements. Interestingly, a study by Doni Jayavelu et al. showed that cancer-associated SNPs are significantly enriched in non-coding regions with function as silencer elements [23]. Huang et al. showed that the rs12631656 variant alters the binding affinity of SOX13 and ARID5B, two repressors in T cells, in a silencer element [40]. In patients with endometrial cancer, the rs2494737 overlaps a silencer element located within the *AKT1* gene [120]. The variant risk allele A creates a new binding site for the YY1 TF, a positive regulator of *AKT1*. These discoveries emphasize the need to deepen investigations into how somatic mutations and SNPs affect silencer elements, holding the potential to unveil a more profound comprehension of their significance in the pathogenesis of cancer.

### Technical approaches to identify and characterize non-coding single nucleotide mutations in GREs

Characterizing non-coding single nucleotide mutations within GREs in cancer requires technology that can precisely identify such mutations and delineate their impact on gene expression. Thus, the techniques can be classified according to the type of information that is generated, ranging from the identification and annotation to the functional validation. While there are diverse approaches available to achieve this objective, it is crucial to carefully weigh the merits and limitations of each approach, which have been summarized in Tables 4, 5, 6, and 7.

**Table 4:** Techniques for identifying novel single nucleotide mutations in GREs

Technique	Advantages	Disadvantages	Ref
WGS	Good minor allele frequency (MAF) estimation. Commercial test available Unpredictable and ultra-rare risk mutations can be detected	Long protocol High computational price Low-frequency genomic coverage	[121, 122]
SNP-arrays	Inexpensive Remarkably precise technology Establish and robust analytical process Cost-effective Large sample sizes	Limited to variants that are common or low in frequency	[121]
Nanopore sequencing	Unpredictable mutations can be detected Long reads Real-time insights No DNA amplification is needed	High cost High error rates Low throughput	[126]
Sanger sequencing	Low price	Previous PCR step SNV cannot be detected if the mutant allelic fraction of the sample is not superior to 20% of the DNA pool	[127]
Digital PCR	A low amount of DNA is needed Easy to use Resistance to PCR inhibitors Feasible quantification	Contamination risk Defined, limited, and single targets Higher price than Sanger sequencing	[183]

**Table 5:** Technological approaches for assessing the impact of non-coding mutations in GREs

Technique	Advantages	Disadvantages	Ref
SNP-SELEX	High sensitivity, throughput, and specificity Reduced background noise	The study design is limited to a small fraction of the human genome SNPs The catalog of TF is anticipated to be expanded	[129]
Allelic imbalance	Advances in the study of germline-somatic alterations Better statistical power. Integration with ATAC-seq data, Regulome-Wide Association Study, and TF motif discovery	Only applied in the study of allelic copy number alterations and not used to study germline regulatory effects	[130]
MPRA	High throughput Quantitative data Fine mapping In vivo assays	Simplified assays Dependence on constructs Resource-intensive Episomal GREs may not mimic chromosomal regulation False-negative and false-positives during experimental design	[131–135]
STARR-seq	Cloning and analysis of libraries are straightforward Decreasing the risk of erroneous barcode-GRE associations	Reduced reproducibility Many sample replicates are needed Preference for sequences that enhance transcript stability	[136–138]
CRISPR Screen	High throughput Endogenous GRE context	The readout is limited by the sgRNA amount Inefficiency and limited scalability Heterogenous cell population so clonal selection is required. Off-target DNA cleavage	[139, 140, 142]
Base editing screen	High throughput Endogenous GRE context	Existing base editors may be incompatible with certain base substitutions Editing window limits sgRNA design	[141]
EMSA	Qualitative and semi-quantitative assay Low cost	False-negative and false-positive results Laborius protocol Purity and specificity TF protein	[143, 144]
ATAC-seq	Can be used in frozen tissues and single cells	Sequencing contamination by mitochondrial DNA High sequencing coverage is required to precisely map factors	[145]

**Table 6:** Technical strategies to profile DNA–Protein interactions

Technique	Advantages	Disadvantages	Ref
ChIP-seq	Many disposable protocols and adaptations High resolution, covers repetitive sequences Genome-wide coverage	Requires a high amount of input cells Needs high-quality antibodies	[146]
CUT&RUN	High sequencing signal-to-noise ratio offering near-base pair resolution Native conditions Low cellular input	Needs high-quality antibodies	[147]
CUT&TAG	Fast Low price Single-cell possibility	Needs high-quality antibodies	[148]

**Table 7:** Methods to study the impact of mutations on chromatin conformation

Technique	Advantages	Disadvantages	Ref
4C	Uncomplicated and goal-oriented	Only examination of chosen genome regions	[150]
5C	Allowing exploring interactions between many fragments, reducing bias Cheaper than Hi-C	Results with lower quality than Hi-C Complicated primer design with only chosen fragments analyzed Limited coverage with missed long-distance interactions	[151]
Hi-C	Genome-wide interactions	Challenging sequencing process and analysis High-priced method. Short-distance interactions are restricted by resolution	[152]
PCHi-C	High-resolution interaction profiles between GREs	Resolution is determined by the restriction enzyme employed for the library production Interactions between GREs on the same restriction fragment are imperceptible to ‘C-type’ assays Millions of cells are required	[153]
ChIA-PET	The higher resolution of protein-bonded long-range interactions	Analysis restricted to the chosen protein	[156]
HiChIP	Genome-wide interaction mapping Less cell quantity requirement Validation of GREs interaction Easy and two-day protocol Enhanced efficiency of DNA contact capture Reduction of false-positive interactions	Limited power to detect nearby genes Difficult computational pipelines	[154]

**Identification of novel single nucleotide mutations affecting non-coding GREs** Detecting somatic SNVs requires analyzing tumor-derived specimens and contrasting them with normal tissues, whereas the SNPs can be determined from virtually any tissue in the subject. Different technologies are available for this purpose. Table 4 provides an overview of these technologies.

Over the past decade, next-generation sequencing (NGS) has led to the discovery of new SNVs in non-coding genomic regions. Whole-genome sequencing (WGS) provides a comprehensive insight into an individual’s genetic makeup. Yet, it faces challenges due to its cost, increased computational expenditures, complex data analysis, and the added burden of multiple tests. Nonetheless, it provides versatility in detecting a wide range of somatic variants, from common to extremely rare, contingent upon sequencing depth [121, 122]. In the next-generation sequencing technologies abovementioned, identifying non-coding variants affecting the TF binding motifs within GREs is highly susceptible to false positives because of the short binding length. Also, analyzing and pinpointing these variants can be challenging due to numerous sequencing chemistry errors that commonly result in many false positive variants [123]. Thus, achieving a balance between eliminating false positive variants (specificity) and retaining true variants (sensitivity) is essential [124]. The progress in computational biology has also radically improved the discovery of novel variants associated with cancer traits. For example, the use of phyloP scores generated from the genomic constraint based on base-pair level conservation across 240 mammals, spanning 100 million years of evolution, can be used for fine-mapping of disease-related non-coding mutations, including cancer [125].

Another technological approach to perform WGS and identify novel SNVs in cancer is Nanopore sequencing, which determines a DNA sequence through the electrical potential perturbations occurring as the DNA strand passes through a pore. It offers distinct advantages, including the generation of long-reads, real-time insights, and direct DNA sequencing without the need for a prior amplification. However, researchers must consider its limitations, such as elevated error rates, lower throughput, high economic cost, and base-calling challenges [126]. Alternatively, SNPs can also be determined using microarray technologies, which utilize reliable genotyping technology, offering a cost-effective approach to identifying risk loci. These arrays rely on established genetic variant reference panels and are inadequate to detect novel or rare disease-contributing SNPs [121]. Finally, once a novel non-coding SNV is identified, the validation on a larger sample cohort can be performed using cost-effective targeted approaches like Sanger sequencing [127] or Digital PCR [128].

**Assessing the impact of non-coding mutations in GREs.** Two main approaches are used to evaluate the effects of germline as well as somatic SNVs in GREs, each with its advantages and limitations. Indirect methods, like whole-genome epigenetic assays, provide a broad overview of a region’s regulatory status but may not pinpoint the impact of a specific genetic alteration [24, 25]. On the other hand, direct methods assess how individual alleles affect gene expression, either in an episomal or native context. However, these direct methods are currently low-throughput and require substantial resources for comprehensive evaluation of non-coding regions, such as repetition of experiments with short DNA fragments. But perhaps one of the main limitations of the latter is the impossibility of assessing the contribution of distal intrachromosomal or interchromosomal regions.

A high-throughput indirect method termed single-nucleotide polymorphisms evaluation by systematic evolution of ligands by exponential enrichment (SNP-SELEX) made estimations of TF relative affinity to predict the effects of non-coding variants [129]. In cancer, the integration of allelic imbalance of chromatin accessibility, TF motif discovery, and Regulome-Wide Association Study help identify potential causal risk variants and elucidate their underlying mechanisms [130].

Regarding direct techniques, the Multiplex Parallel Reporter Assays (MPRA) is based on the introduction of a plasmid construct into a cell containing a reporter gene (luciferase or green fluorescent protein), a promoter, and the mutant GRE candidate. These assays measure changes in luciferase activity or GFP expression to identify whether the mutation induces an activation or inactivation of the gene expression [131]. These approaches have been used in various studies of non-coding mutations in GREs, both in in vitro [131–133] and in vivo models [134, 135]. A specific application of MPRA is the self-transcribing active regulatory region sequencing (STARR-seq) approach, which quantifies the activity of multiple non-coding mutations simultaneously [136]. The STARR-seq method has been useful in systematically assessing the impact of non-coding mutations on GRE function [137, 138].

A primary drawback of these approaches is their inability to effectively evaluate the functional impact of the mutation within the native genomic context. To address these concerns, genome editing techniques provide a more physiologically relevant method for assessing the impact of non-coding mutations on tumor development. One promising approach involves the use of CRISPR and base editing screens with a phenotypic readout achieved by employing a single-guide RNA (sgRNA) dropout [139–142]. However, in cases where the target region is larger, leading to cellular heterogeneity, clonal selection may be necessary. Alternatively, protein binding assays, such as the Electrophoretic Mobility Shift Assay (EMSA), can be employed to elucidate the molecular functions of non-coding mutations. In an in vitro setting, DNA probes are exposed to antibodies targeting the candidate transcription factors to assess the binding affinity of different alleles surrounding a candidate mutation [143]. For unbiased techniques, DNA-affinity pulldown followed by mass spectrometry offers a valuable option [144].

In parallel, ATAC-seq (Assay for Transposase-Accessible Chromatin using sequencing) is a complementary and cost-effective approach that can determine the status of GREs based on chromatin accessibility. A hyperactive Tn5 transposase inserts sequencing adapters into open chromatin regions, which are subsequently subjected to NGS [145]. However, accessibility alone cannot unveil the nature of the GRE and the functional impact of single nucleotide mutations. This prompts the combination of ATAC-seq data with the mapping of histone marks defining promoters, EEs, and IEs, respectively. Table 5 summarizes each strategy, along with its benefits and drawbacks.

**Techniques to identify DNA–Protein interactions**. Since non-coding single nucleotide mutations can alter TFBS sequences influencing the ability of a TF to bind DNA, various approaches are employed to study changes in protein-DNA interactions. These techniques involve ChIP-seq (Chromatin Immunoprecipitation), CUT&RUN (Cleavage under targets and release using nuclease), and CUT&TAG (Cleavage Under Targets and Tagmentation). Table 6 provides a quick overview of these technical strategies.

ChIP-seq is an antibody-based technique that involves crosslinking between DNA–protein complexes, chromatin shearing, and antibody pulldowns for the studied factor [146]. The precipitated DNA fragments are then purified and sequenced or quantified by real-time PCR. However, ChIP-seq can be challenging, particularly when studying target proteins that are part of multiprotein complexes or do not directly interact with DNA, and due to variability introduced during sonication.

To address these challenges, novel techniques have emerged. CUT&RUN employs a recombinant Protein A-MNase fusion construct that binds to the factor of interest’s primary antibody and cleaves DNA around TFBS, generating small fragments for sequencing or real-time PCR [147]. Another innovative method, CUT&Tag, utilizes pA-Tn5 carrying sequencing adapters to generate DNA amplicons for tagmentation-based sequencing [148].

**Techniques to unveil the impact of non-coding mutations on chromatin conformation.** Massive alterations in TADs and chromatin conformation, due to somatic SNVs or pathological SNPs, can be assessed using a variety of techniques. Chromatin conformation methodologies provide essential validation for the impact of non-coding mutations in GREs that affect chromatin looping; nevertheless, their limited throughput makes them less suitable for variant screening [149]. Despite this limitation, it is important to highlight their utility in establishing connections between GREs and specific target genes. Additionally, they can enhance the scope of GWAS by elucidating functional links between non-coding point mutations and GRE activity [121]. Among these techniques, we find 4C (Circular Chromosome Conformation Capture), 5C (Chromosome Conformation Capture Carbon Copy), Hi-C, Promoter Capture Hi-C (PCHi-C), HiChIP, and ChIA-PET. All these methods used for unveiling the impact of non-coding mutations on chromatin conformation are displayed in Table 7.

In 4C, a circularization step is performed to screen physical interactions between chromosomes associated with the genomic region of interest. Subsequently, target genes are amplified to identify genome-wide interactions [150]. On the other hand, 5C involves the relegation of DNA fragments from crosslinked cells to promote ligation between cross-linked interacting DNA fragments, followed by ligation-mediated amplification and sequencing of the target fragment [151]. In Hi-C, after DNA digestion is completed, the ends of the fragments are labeled with biotinylated nucleotides for ligation and reversal of crosslinks, followed by sequencing using paired-end sequencing [152]. Additionally, PCHi-C allows the genome-wide detection of distal promoter-interacting regions using Hi-C libraries enriched in promoter sequences. This is achieved by selecting biotinylated RNA baits that are complementary to promoter-containing restriction fragments. The objective is to capture promoter sequences and their interacting GREs, thereby increasing the number of reads covering promoter regions and improving the sensitivity of the technique for these regions [153]. HiChIP has recently been introduced by Mumbach MR, et al., incorporates in situ Hi-C and transposase-mediated on-bead library construction with a robust, reproducible, and two-day protocol [154]. In HiChIP, long-range DNA interactions are initially formed within the nucleus before lysis, reducing the potential for false-positive interactions [155] and significantly enhancing the efficiency of DNA contact capture.

Finally, ChIA-PET takes a different approach to explore chromatin conformation by crosslinking DNA–protein complexes with formaldehyde in the nucleus, followed by sonication-induced breaks. After reversing the crosslinking, protein complexes are digested, and DNA fragments are extracted for sequencing [156]. The sequencing reads are then aligned and scrutinized to unveil long-distance interactions between TFs.

### Challenges and future perspectives in the research of non-coding mutations with functional impact on GRE activity

Understanding the intricate landscape of non-coding mutations within GREs is pivotal for deciphering their roles in cancer initiation and progression, and their potential diagnostic and therapeutic implications. The main challenge lies in precisely pinpointing the genomic coordinates of the mutation and discerning the impact on the affected GRE [157]. However, an unsolved issue is determining when these non-coding mutations occur during tumor development and progression. Recent advances in single-cell technology have started to identify sub-stoichiometric alterations and their possible contributions to cancer providing a tool to potentially predict the timeline of occurrence [158].

The limited understanding of the non-coding genomic space has led to disparities in variant annotations across various databases, resulting in divergent predictions. Therefore, standardizing non-coding mutation annotation is an imperative step forward in this field [159]. Moreover, due to the lack of experimental data, many annotations in these databases rely on *in-silico* predictions. While international collaborative efforts have yielded proficient tools for variant calling, such as GATK (https://gatk.broadinstitute.org/hc/en-us), the extensive annotation of the non-coding genome remains an ongoing challenge. Additionally, the activity of GREs may vary with the tissue and site of origin, as well as the intrinsic heterogeneity present within cells, especially in the context of cancer, which brings additional challenges to predicting functional impact and outcomes. The coexistence of multiple genes within the same genomic region further complicates the endeavor of defining driver non-coding mutations on GREs.

The next major challenge involves translating these non-coding mutations into their causative roles in altering oncogenic networks. Researchers employ various experimental and in silico methods to characterize potential pathogenic non-coding mutations. Due to the insufficient experimental data, current in silico approaches utilize multiple machine learning and mathematical modeling to make predictions with available published data [160, 161]. For example, TURF [162] and GRAM [163] are computational tools that integrate various layers of information to prioritize non-coding regulatory variants across the human genome. Important databases such as the Ensembl project (https://www.ensembl.org) include the Ensembl Variant Effect Predictor, a robust toolset for analyzing, annotating, and prioritizing genomic variants in both coding and non-coding regions [164]. Fu et al. created FunSeq2, a computational framework designed to annotate and prioritize noncoding mutations by integrating extensive genomics and cancer datasets within a customizable context [165]. Additionally, the Chromatin-Chromatin Spatial Interaction (CCSI) database displays chromatin interactions along with associated genes, EEs, and SNPs, offering comprehensive interaction maps and providing an analysis pipeline for annotating interactions [166]. GWAS4D (https://mulinlab.org/gwas4d) is a free web server that systematically analyzes genetic variants that could influence GREs by integrating annotations from cell type-specific chromatin states, epigenetic modifications, sequence motifs, and cross-species conservation [167]. Furthermore, Li et al. developed OncoBase, a valuable resource for the functional annotation of non-coding regulatory regions and for systematically benchmarking the regulatory effects of embedded non-coding somatic mutations in human carcinogenesis [168]. Lee PH et al. provide a comprehensive review of existing data resources and advanced analytical methods for aiding the *in-silico* prioritization of non-coding mutations [169]. Nonetheless, it’s crucial to acknowledge that each bioinformatic approach has its limitations, and variability exists between them [170].

While numerous assays have been conducted in cancer cell lines, they often overlook cellular diversity and physiological context. To address these limitations, researchers are turning to animal models and the raising application of genome editing techniques. For example, these approaches have been employed to create mice with mutations in the *TERT* promoter region, providing insights into non-coding TERT mutations detected in melanomas [171]. For EEs, although in vivo studies specific to cancer are still lacking, studies related to type 2 diabetes [172] and orofacial clefting [173] exist in the zebrafish model and polydactyly [174] and neuropsychiatric disorders [175] in mouse models. In the case of SEEs, Cui, S et al. deleted the EphA2-SEE in a xenograft model, which is present in various tumor types, effectively suppressing tumor proliferation [176]. Finally, in the context of IEs, mouse models incorporating mutations at CTCF binding sites were employed for developmental studies [177, 178], although their relevance to cancer research is limited. Collectively, these studies suggest that rectifying non-coding mutations within GREs offers a promising avenue for cancer therapeutics, even though in vivo research faces throughput limitations.

Equally important is the urgent need to translate non-coding GRE mutations into clinical significance, which could reshape cancer genomic medicine. Continuous advancements in CRISPR/Cas and base editing technologies are pivotal in this endeavor. For instance, in patients with β-thalassemia, an erythroid-specific EE within BCL11A contains numerous non-coding mutations that suppress γ-globin expression and fetal hemoglobin in erythroid cells [179]. Utilizing CRISPR/Cas9, researchers disrupted GATA1 binding sequences within the BCL11A EE, ultimately restoring γ-globin synthesis and fetal hemoglobin production in patients with β-hemoglobinopathies [180, 181]. Currently, there are no similar clinical trials applied to cancer using the CRISPR system. However, it is important to consider a series of potential limitations such as the presence of pre-existing immunity against CRISPR components restricting the safety and feasibility of in vivo delivery [182].

## Conclusions

Recent advancements in sequencing techniques have significantly enriched our understanding of the impact of germline and somatic non-coding mutations in cancer. These alterations can occur in various non-coding gene regulatory regions of the genome, including promoters, EEs, IEs, and silencer elements. Notably, single nucleotide mutations within these regions can disrupt TFBSs, thereby altering TF recognition on gene regulatory elements. Consequently, this disruption can lead to a perturbation in the gene expression networks, ultimately resulting in an imbalanced expression of tumor suppressor genes and oncogenes.

Different techniques are available for the detection and functional inference of non-coding single nucleotide mutations in cancer. Both in vitro and in vivo models can be employed to assess the targetability of candidate variants, which, in turn, may inform the development of novel drugs and gene therapy strategies, or the development of prognostic or predictive biomarkers.

Despite the challenges posed by technical limitations, population heterogeneity, and inconsistencies in SNV annotations, recent research findings indicate a substantial impact of non-coding alterations on cancer development and progression. It is, therefore, essential that ongoing research efforts continue to elucidate the intricate links between non-coding mutations in gene regulatory regions and pathology. Moreover, the translation of this knowledge from laboratory research to clinical application is utterly important, specifically for aggressive forms of cancers that still do not have effective treatments. Thus, research in this field may ultimately fill the gap between benchside discoveries and bedside patient care.

## Summary

- Genomic alterations in non-coding regions, including somatic single nucleotide variations (SNVs) and single nucleotide polymorphisms (SNPs), can impact gene regulatory elements (GREs) and play a role in human disorders, including cancer.
- Sequencing technology advances have revealed that over 90% of mutations in cancer are in non-coding genome regions.
- Oncogenic somatic SNVs and SNPs within GREs can disrupt transcription factor binding sites (TFBS), leading to alterations in epigenetic mechanisms, including changes in chromatin accessibility and DNA methylation.
- Cutting-edge and emerging high-throughput sequencing technologies allow the identification and cataloging of non-coding genomic alterations, thereby enabling a comprehensive exploration of the intricate landscape of genetic mutations within GREs.
- Additional challenges lie in the massive data interpretation and understanding of the functional impact of these single nucleotide mutations on gene regulatory elements.
- Understanding these alterations is crucial for identifying new theranostic biomarkers to add a new layer of information to improve the clinical management of patients with cancer.

## Data Availability

Not applicable.
